# Supplementary material for: The interactome of intact mitochondria by cross-linking mass spectrometry provides evidence for coexisting respiratory supercomplexes
Source: Mol Cell Proteomics. 2017 Dec 8;17(2):216–32. doi: 10.1074/mcp.RA117.000470 (PMC5795388; doi:10.1074/mcp.RA117.000470)
Supplement: Supplemental Data [file supp_RA117.000470_133922_0_supp_23978_fzffwf.zip › spectra_annotation/mito_DR_spectra_annotation/61-1-8-1-26-1.pdf]

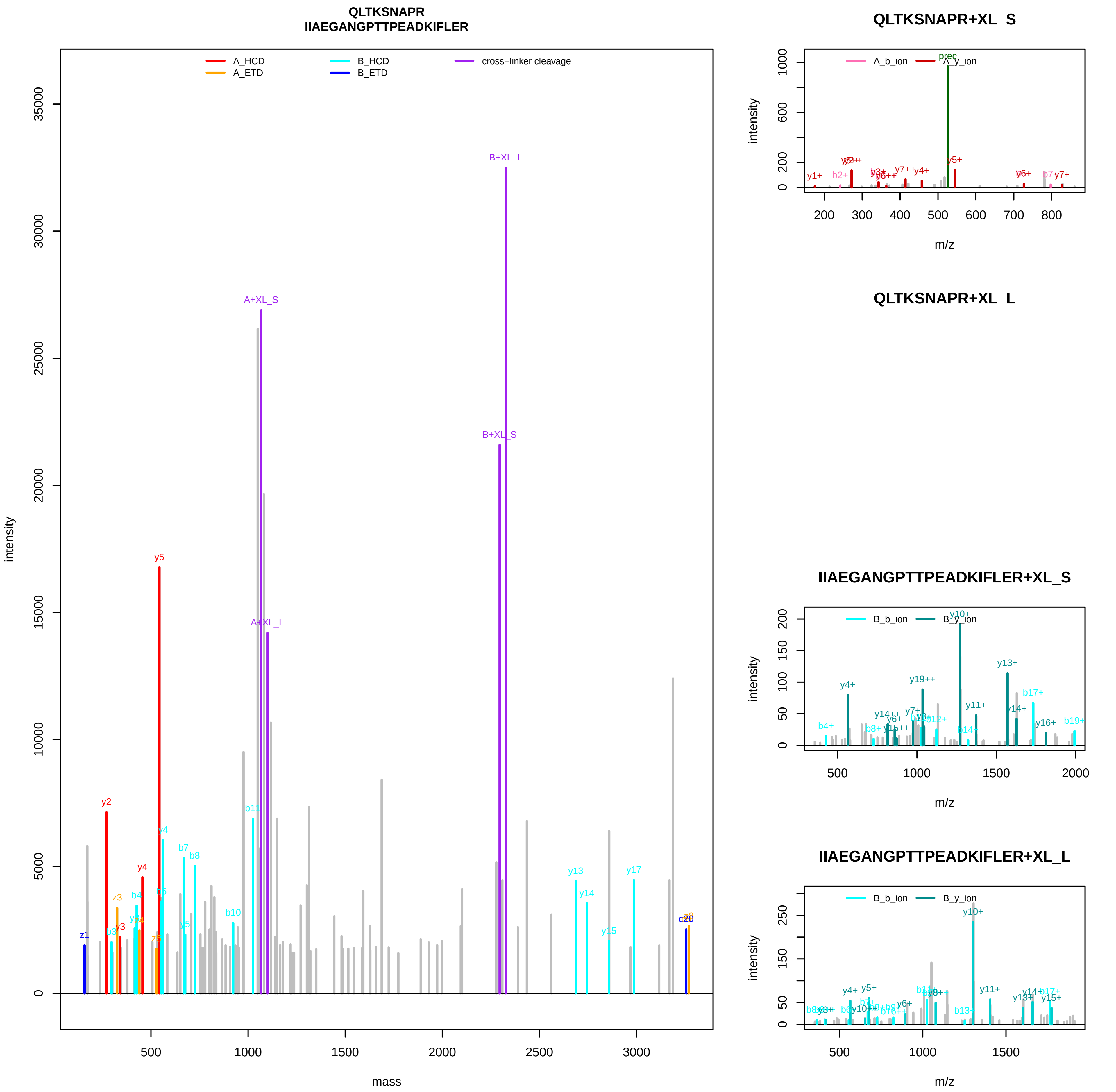
QLTKSNAPR
IIAEGANGPTTPEADKIFLER
A_HCD
A_ETD
B_HCD
B_ETD
cross−linker cleavage
intensity
mass
B+XL_L
A+XL_S
B+XL_S
A+XL_L
y5
y2
y4
z3
y3
b4
b3
z1
y4
b7
b8
b6
y5
b10
b11
y13
y14
y15
y17
c20
0
5000
10000
15000
20000
25000
30000
35000
500
1000
1500
2000
2500
3000
QLTKSNAPR+XL_S
A_b_ion
A_y_ion
prec
y1+
b2+
y5+
y6+
y7+
y4+
m/z
200
300
400
500
600
700
800
QLTKSNAPR+XL_L
IIAEGANGPTTPEADKIFLER+XL_S
B_b_ion
B_y_ion
y10+
y13+
y19++
y4+
b17+
y11+
y14+
b4+
y16+
b19+
IIAEGANGPTTPEADKIFLER+XL_L
y10+
y11+
y4+
y5+
y6+
y13+
y15+
b17+
